# Interviews with primary care physicians identify unmet transition needs after ICU

**DOI:** 10.1186/s13054-022-04125-7

**Published:** 2022-08-15

**Authors:** Katrina E. Hauschildt, Rachel K. Hechtman, Hallie C. Prescott, Leigh M. Cagino, Theodore J. Iwashyna

**Affiliations:** 1 Veterans Affairs Center for Clinical Management Research, HSR&D Center of Innovation, Ann Arbor, MI USA; 2 Division of Pulmonary and Critical Care Medicine, Department of Internal Medicine, Johns Hopkins School of Medicine, Baltimore, MD USA; 3 Division of Pulmonary and Critical Care Medicine, Department of Internal Medicine, University of Michigan, Ann Arbor, MI USA

**Keywords:** Care transitions, Primary care, Communication, Financial toxicity, Post-ICU care

## Abstract

**Aim:**

We sought to explore unmet needs in transitions of care for critical illness survivors that concern primary care physicians.

**Findings:**

Semi-structured interviews with primary care physicians identified three categories of concerns about unmet transition needs after patients’ ICU stays: patients’ understanding of their ICU stay and potential complications, treatments or support needs not covered by insurance, and starting and maintaining needed rehabilitation and assistance across transitions of care.

**Conclusion:**

Given current constraints of access to coordinated post-ICU care, efforts to identify and address the post-hospitalization needs of critical illness survivors may be improved through coordinated work across the health system.

**Supplementary Information:**

The online version contains supplementary material available at 10.1186/s13054-022-04125-7.

## Introduction

Access to specialized post-ICU care or follow-up remains limited in the USA [1–3]; most critical illness survivors will receive follow-up by a primary care provider (PCP) [4]. We hypothesized that experienced primary care physicians may be aware of gaps in ICU discharge processes, and therefore, we conducted interviews with such physicians to identify what they felt were common gaps that could be improved outside of specialized post-ICU settings.

## Methods

We conducted semi-structured interviews about hospital discharge summaries with 14 primary care providers in Internal Medicine and Family Medicine at a large US academic institution between September 2020 and April 2021 [5]. Our study was classified as exempt by the University of Michigan Institutional Review Board. Our interview guide was developed using existing literature on discharge transitions. Participants were identified using snowball sampling until meeting Malterud’s criteria [6] for “information power.” By recruiting a highly informed sample and achieving high quality dialogue, we achieved sufficient information power for our aims in 14 interviews. We used a modified Rigorous and Accelerated Data Reduction (RADaR) [7] process to identify critical issues described by participants. In another paper [5], we describe results focused on improving hospital discharge summaries. This letter focuses on patients’ unmet transition needs that also arose during these interviews (see Additional file 1: appendix for more information).

## Results

Primary care physicians, described in Table 1, raised three broad concerns about unmet transition needs after ICU stays: patients’ understanding of their ICU stay and potential complications, treatments or support needs not covered by insurance, and starting and maintaining needed rehabilitation and assistance across transitions of care.

**Table 1:** Demographics of sample

*Gender*	
Men	2
Women	12
*Race*	
White	8
African American, Middle Eastern, Asian	6
*Board certifications*	
Internal Medicine	11
Family Medicine	3
Pediatrics	2
*Years in practice*	
1–5	3
6–15	5
16 +	6

### Patients’ knowledge of ICU course

Primary care physicians spoke of patients’ confusion about their ICU stays and new limitations resulting from critical illness. Primary care physicians felt this reflected patients’ limited participation in their care in the ICU due to illness and sedation, the complexity of critical illness, as well as the limitations of current discharge information provided to patients, which they felt was usually not ICU specific.

*02: Most patients have no idea what happened to them.*

*04: Patients—the longer the hospitalization, or the more complex—they just don’t know what was going on, they don’t know all the people that they’ve seen.*

*10: The patient rarely remembers anything that happens in the ICU, and family members, even if they remember things, don’t often understand them.*

Primary care physicians desired to help patients process their ICU stay and answer questions but felt overwhelmed when patients knew nothing about their hospitalization or expectations for when they could return to work or possible post-intensive care syndrome (PICS) issues, which can develop after hospitalization.

*01: [Patients] are only half listening some of the time, or can only take in half of it, and I think there’s stuff that’s really practical: When can I go back to work? When do you think I’ll be off of oxygen? What should I look out for? What is bad?*

*07: I’m sure that the provider said something about it, but it did not sink in with the patient… It’s hard to know exactly what really worked in the education in the hospital when an ICU visit is so traumatic!*

### Needs not covered by insurance

Primary care physicians shared varied examples of care and treatment prescribed by inpatient teams which were not covered by patients’ insurance or public benefits in the US context. While not limited to patients following critical illness, these problems impacted outcomes after critical care. Primary care physicians specifically highlighted ongoing wound care needs, home modifications, and home care because of new disability—the sorts of problems often targeted by ICU mobility programs.

*08: They’re at home, which is good… they want to be at home, but then they don’t have the support… to have a daily person to come and help them… this is all a result of their hospital stay… that’s really the debility that leads to the need for more support and the inability to pay to have people support them in their homes as a result of a prolonged hospital…especially a prolonged ICU stay.*

*05: They do have a lot of cost concerns about dressings, because those are not easily paid for… they go home from the hospital with like a 4 or 5-day supply, and then the family assumes that we’re just going to be able to keep giving them, but I don’t have a supply in the clinic of this.*

Primary care providers also linked these unmet care needs to patients’ broader ability to recover and maintain independence after critical illness and described feeling overwhelmed addressing complex recovery needs in time-limited follow-up appointments.

*11: They’ll go home with a wheelchair, and there’s no ramp… I feel the most helpless when it’s—you’d love to be able to get them their home modified, so they could stay in it. Because when they move, they lose their support, they lose their neighbors, they lose their pet, and then it seems like a lot of things spiral.*

*07: I think that sometimes as PCPs we feel like people come to us and we might not have what we need to do the best we can for them, and that’s really frustrating, like we’re trying our best, but we need help … sometimes it’s help from like social work, or just financial resources, or community resources, or just a better system.*

### Starting and maintaining needed rehabilitation and assistance across transitions of care

Primary care physicians noted that a lack of information about patient’s functional status at discharge combined with additional transitions of care after discharge to skilled nursing facilities (SNFs) and rehabilitation facilities could lead to unmet assistance needs, delayed occupational and physical therapy care, and potentially additional deterioration in status.

*11: The man could have come home completely dependent, and I don’t find out about it until the woman hurts herself trying to pick him up, and you’re like, “How long has he—?” [they reply] “Well, he came home like this.”*

*01: Sometimes it is not clear… what you’ve already planned as a transition, meaning—do you already have homecare coming? Is the person going to a SNF? … And like, homecare—is it just home nursing? Because sometimes then you’re like, “Add some PT [physical therapy] on there even before I see the person, just because I know them.”*

*09: The one thing … that comes up actually a lot … is their functional status. I don’t get a sense of… what were the decisions that played into their placement … instead, I’m digging through PT notes… PT notes are very templated, and I feel like are driven by insurance coverage—what insurance needs—so when I read them I don’t understand what the person can actually do.*

Primary care providers felt that placements in SNF and rehabilitation facilities were particularly common among survivors of critical illness and often focused on improving functional status. Unfortunately, patients’ time in post-hospital facilities did not help primary care physicians in follow-up, because discharge information from these facilities was described as very limited or non-existent—one described it as a “black box”.

*12: I don’t get anything about what happened in the rehab place.*

*I: Do you get a discharge summary from the post hospital setting? 08: No, 99% of the time, no… Like, I hope that there’s like a family member that comes to tell you, and [has] some reasonable understanding, but it’s just a nightmare, and that’s an area for massive improvement.*

## Conclusion

Our findings reveal that primary care providers have concerns about post-critical illness transitions of care that could be partially remediated by actions of ICU clinicians even absent access to post-ICU clinics [1, 2].

Primary care physicians wanted better information sharing with patients and families about patient’s ICU course and potential post-ICU complications [8, 9], particularly when family involvement in the ICU was restricted [10–12]. Narrative, written, plain language information about a patient’s ICU stay, available after discharge, which can be discussed with outpatient providers, may alleviate some of these problems [13, 14].

Our respondents’ concerns also contribute to the literature identifying financial issues after critical illness [15–17]. Insuring adequate supplies and care may be important targets for recovery [18, 19]. Social work follow-up or social welfare consultation after hospital discharge may help alleviate potential financial burdens in some systems [18, 20], and such referrals may help alleviate burdens on primary care physicians trying to manage other recovery needs in limited appointment time.

Our findings are exploratory in nature. There are likely additional transition-related issues that other primary care providers working in other health systems or geographic regions may identify; interviews with patients may also reveal additional needs. Further work is needed to develop and test interventions to mitigate transition-related issues. As we confront an increased number of critical illness survivors from COVID-19 and the current constraints of access to coordinated post-ICU care or COVID-specific follow-up, efforts to address post-ICU syndrome and Long COVID effects will demand coordinated work across the health system.

## Supplementary Information

**Additional file 1.** Methods Appendix.

## Data Availability

The data generated and analyzed during the current study are not publicly available as full transcripts are considered identifiable data by VA HSR&D and cannot be publicly available.

## Abbreviations

PCPs: Primary care providers
RADaR: Rigorous and accelerated data reduction
PICS: Post-intensive care syndrome
SNFs: Skilled nursing facilities
PT: Physical therapy
