# Supplementary material for: Interviews with primary care physicians identify unmet transition needs after ICU
Source: Crit Care. 2022 Aug 15;26:248. doi: 10.1186/s13054-022-04125-7 (PMC9376575; doi:10.1186/s13054-022-04125-7)
Supplement: Supplementary file 1 — Additional file 1. Methods Appendix. [file 13054_2022_4125_MOESM1_ESM.docx]

**Interviews with primary care physicians identify unmet transition needs after ICU**

**Methods Appendix**

This paper is the result of additional theme development that took place as part of a semi-structured interview project aimed at identifying what information primary care physicians in our health system wanted to know about patients’ who had ICU stays during their hospitalization, and to what extent current hospital discharge summaries aligned with their needs. The primary manuscript was published in Critical Care Explorations in June 2022 (PMID: 35702352).

We conducted 14 semi-structured interviews between September 2020 and April 2021 with primary care physicians in our academic health system. We drew on Malterud’s criteria for “information power” to guide a snowball sampling approach. We contacted acquaintances providing primary care in the departments of internal medicine and family medicine by email and invited them to participate. Further participants were recruited by asking during these interviews if they knew anyone else whom we should interview about this topic. These participants had specific expertise: treating patients with ICU stays after hospital discharge, and all had been primary care providers for at least 3 years (median 10.5 years). More detail about participants is available in Table 1 of the manuscript. Our interviews produced high-quality dialogue for two reasons: our experienced participants and an interview team with complementary skills and training: Dr. Hechtman is also an inpatient and outpatient provider in the same health system, and Dr. Hauschildt is a PhD-trained medical sociologist with substantial qualitative interviewing experience. Response saturation regarding our primary aims was achieved.

A modified version of Rigorous and Accelerated Data Reduction (RADaR) was conducted by Drs. Hauschildt and Hechtman, in consultation with Dr. Iwashyna. The RADaR technique offered several advantages: it could be used without specialty software (any spreadsheet program will work); it allowed us to condense data quickly while offering flexibility within that process; it was easy to learn, and it was easy to follow other fellow researchers’ processes of reduction across tables when sharing documents back and forth. We first organized interview text by respondent and interview question and then developed reduced tables to list specific data elements mentioned by participants as well as thematic memos with example quotations. This combination of methods allowed us to begin representing key information in different ways throughout the analysis process (lists, memos, etc.).

There is one caveat to the use of “information power” in the identification of additional themes of interest beyond the primary aims, and that is the question of saturation. We did not have specific questions about unmet needs, but rather found stories presented by primary care providers about the discharge and follow-up process consistently revealed patterns of unmet needs. Because unmet patient needs were not within the initial aims of our study, these results are inherently exploratory.
